# Functional iron status of chronic kidney disease patients at the University of Ilorin Teaching Hospital, Ilorin, Nigeria

**DOI:** 10.4314/ahs.v22i3.77

**Published:** 2022-09

**Authors:** Emmanuel O Sanni, Hannah O Olawumi, Idayat A Durotoye, Timothy O Olanrewaju, Abiola S Babatunde, Olasunkanmi A Shittu, Sikiru A Biliaminu, Khadijat O Omokanye, Mutiat Kehinde Ogunfemi, Olabisi O Akinwumi, Dapo S Oyedepo, Ayodeji M Adepoju

**Affiliations:** 1 Department of Haematology, Faculty of Basic Clinical Sciences, Nile University of Nigeria, Abuja, Nigeria; 2 Department of Haematology, Faculty of Basic Clinical Sciences, University of Ilorin, P.M.B 1515, Ilorin, Nigeria; 3 Department of Nephrology, Internal Medicine, University of Ilorin Teaching Hospital, P.M.B 1459, Ilorin, Nigeria; 4 Department of Chemical Pathology and Immunology, Faculty of Basic Medical Sciences, University of Ilorin, P.M.B 1515, Ilorin, Nigeria; 5 Department of Haematology and Blood Transfusion, University of Ilorin Teaching Hospital, P.M.B 1459, Ilorin, Nigeria; 6 Department of Hematology and Blood Transfusion, Kwara State General Hospital, Ilorin, Nigeria

**Keywords:** Chronic kidney disease, functional iron status, anaemia

## Abstract

**Background:**

Functional iron deficiency has been found to be a common cause of poor response to erythropoiesis stimulating agents in anaemic patients with chronic kidney disease (CKD).

**Objectives:**

Assess the functional iron status of patients with chronic kidney disease.

**Methods:**

This was a hospital based cross sectional study. The study subjects were chronic kidney disease patients with age and sex matched healthy controls. Full blood count, serum ferritin, soluble transferring receptor, C-reactive protein, serum iron and total iron binding capacity were measured in the patients and healthy controls.

Data was analyzed with statistical package for the social sciences software version 22.0. And the level of statistical significance was set at p. value < 0.05.

**Results:**

The mean ± SD of the age of patient with CKD was 55.0 + 15.4 years, while that of controls was 52.7 + 13.6 years. The mean serum ferritin, serum iron, TIBC and CRP were significantly higher in patients compared with controls (p<0.001, 0.023, <0.001 and 0.001) respectively. Functional iron deficiency was seen in 19.5% of patients with CKD.

**Conclusion:**

The predominant form of iron deficiency in our study was functional iron deficiency.

## Introduction

Chronic kidney disease (CKD) constitutes a major health challenge to patients and health institutions all over the world, with its impacts being felt more in developing countries like Nigeria.^1^ The health care cost attributed to CKD treatment is enormous due to the complications that usually manifest in late stages of the disease.^1^,^2^ One of the complications of CKD is anaemia which is associated with poor outcomes.^1^ Patients with CKD often needs blood transfusions and frequent hospital admissions along-side maintenance dialysis. The treatment of anaemia caused by CKD usually require the use of erythropoiesis stimulating agents (ESA), which is costly and can be associated with development of thromboembolic events and red cell aplasia.^3^

Poor response to ESAs in patients with CKD is a major challenge in the treatment of anaemia, making it difficult to achieve an expected haemoglobin concentration.^4^ However, functional iron deficiency (FID) has been found to be a major cause of the poor response to ESAs. Evaluation of iron status has mainly focused on the level of iron stores, i.e. serum ferritin. Low levels imply absolute iron deficiency. However, storage iron may not be available for utilization.^5^ The assessment of functional iron status in patients with CKD will provide data that will influence the provision of comprehensive and effective management of anaemia in patients with CKD. Early detection and correction of FID in patients with CKD will therefore improve response to ESAs and this will ultimately lead to improvement in cardiovascular status and overall quality of life of patients with CKD

The aim of this study was to assess FID in patients with CKD in our environment. The results from this study will help in understanding the contribution of FID to the anaemia of CKD and will add to existing knowledge that will be of value in the overall management of patients with CKD.

## Methodology

### Study Design

This study was a hospital-based cross-sectional study.

### Study Population

The study sample size was calculated using the Fisher's formula. One hundred and thirteen CKD patients at different stages of the disease who were attending the Nephrology Clinic at the University of Ilorin Teaching Hospital and 113 age- and sex- matched healthy controls recruited among patient's relatives, medical and nursing students as well as the staff of the hospital. The study period was between June and November 2017.

### Inclusion Criteria

Patients with established cases of CKD (eGFR < 60ml/min/1.73m^2^ for ≥ 3 months), aged 18 yrs and above were recruited for the study. Healthy volunteers who gave consent were also recruited. The eGFR was calculated using the chronic kidney disease epidemiology collaboration (CKD-EPI).

### Exclusion Criteria

Chronic kidney patients with haematological disorders eg sickle cell disease and those with active infections such as tuberculosis, HIV and hepatitis were excluded from the study.

## Methods

### Study Tools

1. Informed consent and some demographic information such as the age, gender, race, level of education were obtained using a study proforma.

2. Some haematological parameters (Full blood count, reticulocyte count) and serum ferritin, soluble transferrin receptor (sTfR), total iron binding capacity (TIBC), percentage transferrin saturation (TSAT), C-reactive protein (CRP) levels of both patients and controls were tested. Full blood count (FBC) was done using a five part automated haematology analyser, Sysmex KX-21 (Sysmex Corporation, Kobe, Japan). Serum Ferritin, TIBC, sTfR and CRP were measured with human Fe (ferritin) ELISA kit, sTfR (human) Taiwan ELISA kit and human CRP ELISA Kit respectively. Serum iron and TIBC were assayed by the colorimetric method using pointe scientific, inc iron/TIBC test kit.

### Ethical Consideration

Ethics clearance was obtained from the Ethics Research Committee of the University of Ilorin Teaching Hospital, Ilorin. An informed verbal and written consent were obtained from the prospective participants.

### Statistical Analysis

Data was analyzed using descriptive and inferential statistics on SPSS software version 22.0. The level of statistical significance was set at p-value < 0.05.

## Results

The mean±SD age of the patients was 55.0±15.4 years, and that of the controls was 52.7±14 years. Majority of the patients and controls were males 72.6% and 74.3% respectively (Table 1.1).

**Table 1.1:** Demographic variables of the study participants

Variable	Subject	Control	Total	χ^2^	*p* value
	n (%)	n (%)	n (%)		
**Age**					
≤ 25	8 (7.1)	7 (6.2)	15 (6.6)	2.727	0.742
26 – 35	10 (8.8)	8 (7.1)	18 (8.0)		
36 – 45	8 (7.1)	13 (11.5)	21 (9.3)		
46 – 55	29 (25.7)	31 (27.4)	60 (26.5)		
56 – 65	31 (27.4)	34 (30.1)	65 (28.8)		
> 65	27 (23.9)	20 (17.7)	47 (20.8)		
Mean ± SD	55.00 ± 15.37	52.73 ± 13.59		1.178^t^	0.240
**Sex**					
Male	82 (72.6)	84 (74.3)	166 (73.5)	0.091	0.763
Female	31 (27.4)	29 (25.7)	60 (26.5)		
**Marital Status**					
Single	11 (9.7)	4 (3.5)	15 (6.6)	2.747^Y^	0.432
Married	95 (84.1)	103 (91.2)	198 (87.6)		
Widow	6 (5.3)	4 (3.5)	10 (4.4)		
Divorced	1 (0.9)	2 (1.8)	3 (1.3)		
**Occupation**					
Trader	42 (37.2)	15 (13.3)	57 (25.2)	29.628^Y^	<0.001*
Civil Servant	26 (23.0)	65 (57.5)	91 (40.3)		
Teacher	3 (2.7)	5 (4.4)	8 (3.5)		
Student	6 (5.3)	3 (2.7)	9 (4.0)		
Retired	4 (3.5)	2 (1.8)	6 (2.7)		
Others	32 (28.3)	23 (20.4)	55 (24.3)		
**Level of education**					
None	18 (15.9)	1 (0.9)	19 (8.4)	33.417	<0.001*
Primary	22 (19.5)	14 (12.4)	36 (15.9)		
Secondary	30 (26.5)	16 (14.2)	46 (20.4)		
Tertiary	43 (38.1)	82 (72.6)	125 (55.3)		
**Tribe**					
Yoruba	100 (88.5)	99 (87.6)	199 (88.1)	1.337^Y^	0.720
Hausa	5 (4.4)	9 (8.0)	14 (6.2)		
Igbo	2 (1.8)	2 (1.8)	4 (1.8)		
Others	6 (5.3)	3 (2.7)	9 (4.0)		
**Religion**					
Christian	46 (40.7)	42 (37.2)	88 (38.9)	0.298	0.585
Islam	67 (59.3)	71 (62.8)	138 (61.1)		

χ^2^: Chi square test; Y: Yates corrected Chi square; t: Independent Samples T test

* *p* value <0.05

**Figure 1.1:**
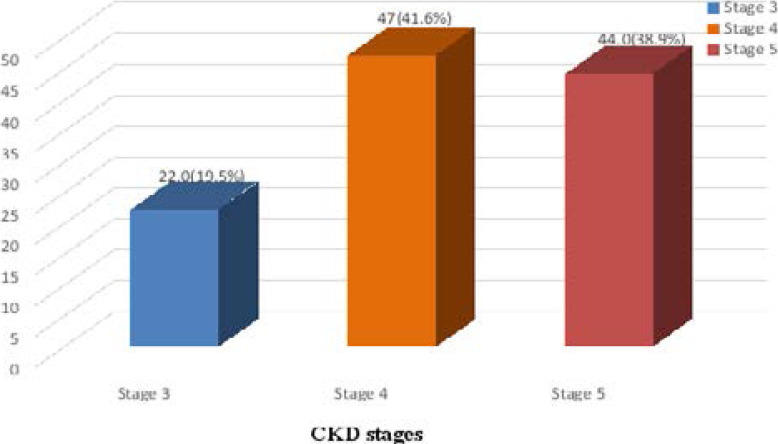
Distribution of the patients with CKD by stages (n=113).

Most of the patients with CKD were in late stages of renal disease, 47 (41.6%) were stage 4 and 44 (38.9%) stage 5.(Fig 1.1).

Hypertension was the commonest underlying cause of CKD, found in 93 patients with CKD (63.7%). The mean haemoglobin concentration (Hb) of patients with CKD was 9.65 ±2.25g/dl while that of controls was 13.62 ±1.45g/dl. P value, <0.001. Table 1.2

**Table 1.2:** Haematological profile of the patients and the control participants

	Patients	Control		
Heamatological parameters	Mean ± Sd	Mean ± Sd	T	*p* value
**Hb (g/dL)**	9.69 ± 2.25	13.62 ± 1.45	-15.608	**<0.001** *
**PCV (%)**	29.02 ± 6.12	40.33 ± 3.89	-16.573	**<0.001** *
**RBC (×10^12^/L)**	3.62 ± 0.92	4.94 ± 0.62	-12.627	**<0.001** *
**MCV (fl)**	80.44 ± 6.65	81.56 ± 4.82	-1.452	0.148
**MCH (pg)**	27.73 ±2.24	28.18 ± 2.30	-1.478	0.141
**MCHC (g/dL)**	33.26 ± 2.89	33.66 ± 1.59	-1.283	0.201
WBC (× 10^9^/L)	6.54 ±2.19	5.66 ± 1.64	3.417	**0.001** *
**PLT count (× 10^9^/L)**	263.67 ±115.45	206.28 ± 70.13	4.516	**<0.001** *
**Reticulocyte count (%)**	0.47 ±0.23	1.26 ±0.36	-19.649	**<0.001** *
**Absolute reticulocyte (/L)**	1.79 ±1.26	6.16 ±2.17	-18.443	**<0.001** *

t: Independent Samples T test

* p value <0.05 (i.e. statistically significant)

Hb, haemoglobin concentration; PCV, packed cell volume; RBC, red blood cell count; MCV, mean corpuscular volume; MCH, mean corpuscular haemoglobin concentration; MCHC; mean corpuscular haemoglobin concentration; WBC, white blood cell count; PLT, platelet.

The mean values of serum ferritin, serum iron, TIBC, TSAT, creatinine and CRP was statistically significantly higher in patients with CKD than control participants. Table 1.3

**Table 1.3:** Serum ferritin, serum iron, TIBC, TSAT, sTfR and CRP levels in patients with CKD and controls

	Patients	Control participants		
Variable	Mean ± Sd	Mean ± Sd	T	*p* value
**Ferritin (ng/ml)**	290.12 ± 161.52	99.46 ± 53.05	11.922	**<0.001** *
**Serum iron (µg/ml)**	122.99 ± 77.29	104.72 ± 34.62	2.294	**0.023** *
**sTfR (nmol/ml)**	18.27 ± 11.38	17.61 ± 6.14	0.544	0.587
**TIBC (µg/ml)**	405.56 ± 147.34	316.75 ± 72.71	5.745	**<0.001** *
**TSAT (%)**	30.72 ± 14.36	32.74 ±6.83	-1.354	0.177
**CRP (µg/ml)**	3.02 ±1.45	1.51 ± 0.95	9.316	**<0.001** *
**Creatinine (µmol/l)**	434.9 ± 368.3	68.7 ± 10.0	10.564	**<0.001** *

t: Independent Samples T test

* p value <0.05 (i.e. statistically significant). sTfR, soluble transferrin receptor; TIBC, total iron binding capacity; TSAT, percentage transferrin saturation; CRP, C-reactive protein.

**Figure 1.2:**
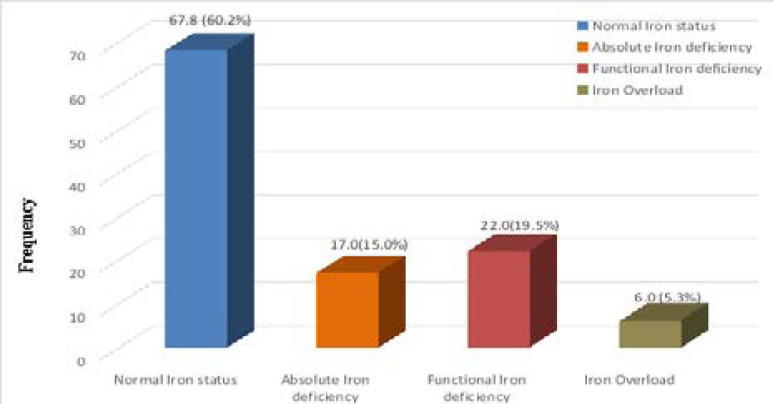
Functional Iron status among patients with CKD

Figure 1.2 shows the iron status among patients with CKD. Normal iron status was seen in 67.8 (60.2%) patients with CKD based on TSAT>20% and ferritin >100ng/ml in predialysis patient with CKD and ferritin >200ng/ml in haemodialysis patients with CKD as recommended by the Kidney Disease Outcomes Initiative (KDOQ1) guideline.3 The guideline recommends the use of serum ferritin and TSAT as indices of iron status to define functional and absolute irn deficiency in anaemic CKD patients.

Absolute iron deficiency was seen in 17 (15.0%), functional iron deficiency was seen in 22.0 (19.5%) while 6.0 (5.3%) had iron overload.

## Discussion

Our study assessed the functional iron status of patients with chronic kidney disease using age and sex matched healthy controls. The prevalence of functional iron deficiency in the patients with CKD in our current study was 19.5% while none was observed among the healthy control participants. Functional iron deficiency was the predominant form of iron deficiency in this study. Our finding is similar to the study done by Suleiman et al at Zaria, Nigeria and Iyawe et al at Ile-Ife, Nigeria where 19.2% and 11% of their CKD patients had FID respectively.^6^,^7^ In contrast to this present study, Lukaszyka et al reported that absolute iron deficiency was more common than functional iron deficiency.^8^ The differences in the pattern of iron deficiency in this present study compared with the latter study may be partly due to the fact that white subjects with a higher proportion (61%) in early stages of CKD were recruited in their study. The relatively lower level of inflammation in the white population compared to the blacks may also be responsible for difference in pattern of iron deficiency.^8^ The mean value of C-reactive protein in this study was 3.02 ±1.45 µg/ml and was statistically significantly higher in the patients compared with control participants with a mean value of 1.51 ± 0.95µg/ml (p value<0.001). This could explain why functional iron deficiency in which its underlying aetiology could be partly attributed to inflammation was higher in the patients than the control participants.

Functional iron deficiency is defined as serum ferritin ≥100ng/ml in predialysis patient with CKD and ≥ 200ng/ml in haemodialyzed patients with CKD and TSAT < 20%.^9^ Functional iron deficiency occurs when there is imbalance between iron requirements of the erythroid marrow and the actual iron supply which can occur when there is retention of iron within body stores often referred to as reticuloendothelial blockade.^10^ The other form of functional iron deficiency occurs when the erythroid marrow is stimulated by ESAs and often occurs during treatments of patients with anaemia of CKD with erythropoietin.^10^ The prevalence of iron deficiency (absolute iron deficiency and functional iron deficiency) in patients with CKD in this study was 34.5%. Suleiman et al in Northern Nigeria reported a value of 35.2%, using similar cut off values for defining iron deficiency as done in this study.^6^ Higher prevalence of 56.1% was reported by Arogundade et al compared to this study.^11^ This higher figure may be due a higher cut off value of serum ferritin of ≥300nm/ml and TSAT of >25% was used to define normal iron status. However, a cut off value of serum ferritin ≥ 100ng/ml for predialysis and ≥ 200ng/ml for haemodialysis patients with CKD and a TSAT of 20–55% was used in this study as recommended by KDIQO1 guidelines.^3^,^12^ In this present study only 60.2% of 113 patients with CKD met these K/DOQ1 targets as stated in the guidelines.^9^ Absolute iron deficiency was found in 15% of patients with CKD in our study which is similar to reports of previous studies.10,13 The national health and nutrition survey suggests that up to 50% of patients with stages ii-v have absolute or functional iron deficiency.^13^ This study revealed that, iron deficiency anaemia is common in patient with CKD.

The prevalence of anaemia in this study was 88.5% in patients with CKD and was significantly higher than that of the controls (21.2%), p value< 0.001. Shittu et al in 2010 reported a prevalence of 94% while Akinsola et al reported a prevalence of 87% among patients with CKD in Nigeria.^14^,^15^ These reported values are comparable with our findings in the current study.^14^,^15^ The reported prevalence of anaemia among patients with CKD in United states of America and Spain were 15.4% and 58.5% respectively.^13^,^16^ Stauffer et a in USA, defined anemia as serum hemoglobin levels ≤12 g/dL in women and ≤13 g/dL in men where as Cases-Amenós et al in Spain defined anaemia as haemoglobin levels <13.5g/dL in males or <12g/dL in females or patients who receive treatment with ESA. However, the reference range used in our study is similar to that of Stauffer at al but lower than that used by Cases-Amenós et al among men. Thus, anaemia is more prevalent among patients with CKD in developing countries than developed countries.10 This may partly be due to the presence of factors that are not directly due to kidney disease which include high burden of infections and malnutrition in developing countries including Nigeria. The mean serum Ferritin in patients with CKD was markedly high (290.12±161.56n/ml) as compared to controls (99.46±53.05 ng/ml), this is similar to that reported by Suleiman et al in Northern Nigeria, Gangaghar et al in India, and Gotlib et al in United States.^6^,^17^,^18^ This is not surprising as ferritin is an acute phase reactant and its expression increases with progression of inflammation in patients with CKD. The mean value of serum iron was statistically significantly higher in patients with CKD (122.99±77.29µg/ml) than that of the controls (104.72±34.62µg/ml) which is similar to report of study by Gupta et al in India.^19^ The findings in this study is at variance with results of study done by Deori et al with reports of lower mean serum iron among the subjects than controls.^20^ Here they believed“the lower mean value of serum iron in the latter study compared with this present study may be due to the fact that patients with CKD on haematinics were excluded in their study”. The mean value of TIBC was statistically significantly higher in patients with CKD compared with control participants, which is similar to previous study done by Deori at al.^20^ This could imply iron deficiency in some patients with CKD in this study moreover, TIBC is a negative phase reactant. The mean values of soluble transferrin receptor (sTfR) in patients with CKD was higher than controls although this was not statistically significant which is similar to report of study done by Toima et al.^21^ The treatment of CKD patients with ESA would increase the total erythroid mass by increasing sTfR. When a patient is found to have an elevated sTfR, the clinician must determine whether it is due to iron deficiency or because patient is on ESA or has increased erythroblast activity.^22^

In this study, the mean level of CRP was statistically significantly higher in patients with CKD when compared with control participants. Our finding is similar to report by Toima et al.^21^ CRP is a member of a class of acute-phase reactants, as its levels rise during inflammatory processes. It is a non- specific but sensitive marker of inflammation. High CRP levels show good correlation with the presence of an inflammatory process.22The mean value of TSAT in patients with CKD was lower than that of the control, although this was not statistically significant. This is similar to reports of studies done by “Oluboyode et al and Suleiman et al in Nigeria.^6^,^23^ However Deori et al reported a statistically significant lower value of TSAT in patients with CKD.20 The difference in this present study compared to the latter study may be that anaemic patients with CKD in this study were on ESAs and iron supplement as part of their treatment protocol as recommended by the Kidney Disease Outcomes Initiative guidelines.^12^ The guidelines recommend all anaemic adult patient with CKD on ESA be placed on iron supplementation. Patients on haematinics were excluded from the study done by Deori et al.^20^

The major causes of CKD in this study were hypertension, diabetes mellitus, and chronic glomerulonephritis. Although in previous studies done in Nigeria, chronic glomerulonephritis and hypertension have been reported to be commoner than DM as a cause of CKD.^24^,^25^ The rising prevalence of DM as a cause of CKD may be due to the rising prevalence of DM in Nigeria, which may be connected with increasing adoption of westernized culture. This study is limited by the small sample size, we suggest further studies involving a large sample size to affirm the outcomes from this study.

## Conclusion

Functional iron deficiency was found to be the predominant form of iron deficiency anaemia in the studied patients. Patients with CKD who have high CRP levels is associated with increased risk of developing functional iron deficiency. We recommend that iron indices(serum ferritin, serum iron, TIBC and TSAT) with inclusion of C-reactive protein should be incorporated into routine investigations of patients with CKD in order to manage, treat, and monitor absolute and functional iron deficient patients as per KDOQI guidelines.^12^ Also, patients with poor response to ESA and Iron therapy should be investigated for functional iron deficiency.
